# A Longitudinal Study of Maternal Postnatal Bonding and Psychosocial Factors that Contribute to Social-Emotional Development

**DOI:** 10.1007/s10578-022-01398-5

**Published:** 2022-07-23

**Authors:** E. Rusanen, A. R. Lahikainen, E. Vierikko, P. Pölkki, E. J. Paavonen

**Affiliations:** 1 Faculty of Educational Sciences, University of Helsinki, P.O. Box 9, 00014 Helsinki, Finland; 2 Public Health and Welfare, Finnish Institute for Health and Welfare, P.O. Box 30, 00271 Helsinki, Finland; 3 Faculty of Social Sciences, University of Tampere, FI-33014 Tampere, Finland; 4 Faculty of Social Sciences, Tampere University, FI-33014 Tampere, Finland; 5 Department of Social Sciences, University of Eastern Finland, P.O. Box 1627, 70211 Kuopio, Finland; 6 Pediatric Research Center, Child Psychiatry, University of Helsinki and Helsinki University Hospital, P.O. Box 400, 00029 HUS, Finland

**Keywords:** Perinatal bonding, Maternal relationships, Social-emotional development, Depression, Stress

## Abstract

In this longitudinal study, we examined how maternal bonding and psycho-social factors are associated with social-emotional problems in two-year-old children. Our data came from a birth cohort from which data were collected at four timepoints: prenatally during the third trimester, and postnatally at 3, 8 and 24 months. The participants were 1,667 mothers, of which 943 (56.6%) returned the questionnaire at each timepoint of the longitudinal study. The Children’s social-emotional problems were examined using the Brief Infant-Toddler Social and Emotional Assessment. According to linear regression analysis, maternal bonding difficulties at three and eight months, maternal expectations of the unborn baby during pregnancy, and maternal relationships within and outside the family were related to social-emotional problems in children of two years of age. The results highlight the importance of screening mothers who already prenatally have bonding problems or mothers who have bonding problems postnatally to provide effective and targeted intervention support.

**Electronic supplementary material:**

The online version of this article (doi:10.1007/s10578-022-01398-5) contains supplementary material, which is available to authorized users.

## Introduction

The mother–baby bond is important for the physical, psychological, and social development of the baby, as some previous studies have reported (1– 6). As pre- and postnatal bonding are related to each other [7–9], they have both been connected to child development. Alhusen et al. reported [1] that the prenatal maternal bond is associated with a child’s communication, gross and fine motor skills, and problem-solving and personal-social skills at the age of 14 to 26 months. According to Behrendt et al. [2], good postnatal bonding and maternal sensitivity when a baby is six to eight months old are associated with less social-emotional behavioral problems (i.e. dysregulation, externalizing and internalizing problems) and with better social-emotional competence at the age of 12 to 16 months. In addition, Le Bas et al. reported [5] in their recent longitudinal study that maternal bonding during pregnancy and at eight and twelve months postpartum were associated with a child’s social-emotional, behavioral, and temperamental development at 12 months of age.

Bonding is also associated with maternal care and the mother’s interaction with the baby. For example, Siddiqui & Hägglöf reported [10] that mother’s good prenatal bonding (in terms of interaction and affection) is associated with more proximal stimulation (referring to touching and kissing, when babies are 12 weeks of age) but not with maternal responsiveness [10]. Respectively, Sacchi et al. reported (6, p. 4) that prenatal bonding is associated with better maternal structuring abilities (i.e., the ability to guide the child’s explorative behavior and maintain emotional contact and availability at the same time) with a four-month-old infant.

There is also theoretical evidence of the importance of an affectionate bond between mother and child for the child’s developmental outcomes. In a good, reciprocal relationship, both the mother-figure and the child value closeness and affectionate interaction and strive to promote it, and distance and expressions of rejection are perceived as painful (11, p. 242). Both parties in a relationship strive for intimacy, and shared affection and joy in their interaction is important (5, p. 9). However, initially the adult is responsible for this closeness, as a child’s ability to maintain closeness develops gradually (11, p. 265–268). According to a systematic review by Le Bas et al. [4] a good mother–baby bond is associated with various positive developmental outcomes in children such as better attachment quality, positive mood, less colic, and better social-emotional development between 0 and 24 months.

However, previous studies have only analyzed a small number of cases or have been cross-sectional. In longitudinal studies, follow-up periods are often relatively short [4–6, 12]. These factors consequently limit research designs in testing hypotheses.

This study adds to this research tradition by presenting findings from a large birth cohort with a follow-up period from the third trimester of pregnancy until the child is two years old. Our study adds to earlier studies in three ways. First, in our research design, the observation period was long, over two years. Second, we studied a large number of families. These factors enabled us to include several types of anticipatory factors (both psychological and social, in addition to measuring bonding several times) that contribute to a child’s social-emotional development.

Several factors can impair the development of maternal bonding. Maternal mood disturbances and perceived stress are related to difficulties in bonding [9, 13]. Behrendt et al. reported [2] that depressed mothers had difficulties regulating their emotions, were less sensitive, and had more problems with postnatal bonding when their child was six to eight months old. According to Mason, Briggs & Silver [14] maternal postpartum depression at two months was negatively related to postpartum bonding at six months, which in turn, was negatively associated with the child’s social-emotional development at six months.

Maternal depression is strongly associated with a child’s psycho-social development. The association between maternal depression and infant social-emotional development has been more extensively studied than its significance for maternal bonding. It is well established that maternal depression impairs an infant’s social-emotional development at 12–24 months of age [2, 15–22], and up till adolescence [20].

Maternal perinatal stress is also considered detrimental to a developing baby. According to Thiel, Eberhard-Gran and Garthus-Niegel [23], stressors close to birth can become a burden to the mother and can have long-term social-emotional and neurobiological consequences for the child’s development. Researchers have reported that maternal perinatal stress and depression (measured at eight weeks and two years after the baby’s birth) are associated with social-emotional symptoms in two-year-olds [23]. Karam et al.’s results [24] were consistent with those of Thiel. They found that maternal and paternal postnatal stress impaired social-emotional and motor development in one-year-olds. Of the prenatal stressors, only mother’s stress was inversely related to a one-year-old’s motor development [24]. Talge, Neal and Glover reported [25] in their review that prenatal stress is widely related to developmental problems, such as emotional and cognitive difficulties, the risk of attentional deficit/hyperactivity, anxiety, and language delay. Parenatal stress can also have long-term negative consequences for a child. Neece et al. found in their longitudinal study [26] that parental stress was associated with behavioral problems at three to nine years of age in both normally developed children and those with developmental delays.

Even though connections between maternal psychological risk factors (such as depression and stress) and a child’s social-emotional development have been extensively studied, we found only a few longitudinal studies that relate pre- or postnatal bonding to infants’ social-emotional development when maternal perinatal depression or stress are taken into account [1, 2, 5, 14]. The study by Alhusen et al. [1] took postpartum depression and maternal avoidant attachment style into account, and the significance of prenatal bonding for a 14–26-month-old child’s development disappeared. In contrast, according to Behrendt et al. [2], better postnatal bonding was associated with better social-emotional competence and less social-emotional problems in children aged 12 to 16 months, even when postnatal depression and maternal emotion regulation were taken into account.

Interactional factors also facilitate good development of maternal bonding. It has been reported that maternal social support is important for bonding to develop well. Both pre- and postnatal bonding have been associated with a mother’s good relationships with adults within and outside the family [9, 13]. According to Shin, Park and Kim [27], support from others is a significant factor associated with maternal sensitivity to babies at six weeks of age.

In our longitudinal study, we investigated whether the pre- and postnatal mother–baby bond, and a mother’s relationships within and outside the family are related to a child’s social-emotional problems at the age of two. In our study, the concept of prenatal bonding refers to prenatal attachment, or maternal expectations of the unborn baby. Because depression and stress have also been found to impair a child’s development (16–19, 21–25), and because they both also impair maternal bonding (9, 28–32), we controlled for these factors. Maternal adult relationships within and outside the family are relevant because they have been associated with bonding (9, 28, 29, 32).

Based on previous studies, we assumed that: (1) weaker prenatal expectations of the unborn baby [1, 3], (2) weaker mother–baby bonding at three and eight months postpartum [2, 4, 5, 14], and (3) a more negative family atmosphere and weaker relationships with adults [14, 28, 32] would be associated with less optimal social-emotional development of two-year-old children.

## Materials and methods

### Sample

This study was based on the CHILD-SLEEP birth cohort, which was set up to study children’s health, development, and sleep quality. The first measurement point was during pregnancy at about 32nd pregnancy week and families were followed-up at about 3, 8, 18 and 24 months after delivery. The sample was collected systematically during 2011–2013 when all maternity clinics (N = 63) in Pirkanmaa Hospital District, southern Finland participated the recruitment.

The parents were recruited to the study in health centers in the target area during their normal pregnancy follow up visits to the maternity clinics. The recruitment was planned to occur approximately at the 32nd gestational week. As many as 120 nurses in 63 maternity clinics participated the recruitment process. The purpose and the protocol of the study were described in detail to the nurses and the study materials were delivered to the maternity clinics. The nurses provided the parents with oral information on the study protocol and an information sheet about the study. All Finnish-speaking families were eligible for the study. If the family decided to take part, they were given the first set of questionnaires and asked to sign the consent forms for participation in the study. The participants received no monetary compensation and they were informed that they could discontinue their participation in the study at any time. The study protocol was accepted by the local ethical committee (R11032/9.3.2011).

The sample is representative of the general population in the area [33]. The prenatal data comprised 1667 families, of which 1421 (85.2%) returned both the prenatal and the three-month postnatal questionnaires, and 1298 (77.9%) returned both the prenatal, and the three- and eight-month postnatal questionnaires. A total of 943 families (56.6%) returned the prenatal and 3-, 8- and 24-month postnatal questionnaires. (Table 1)

**Table 1:** Description of sample

	*N*	*%*
**Age of mothers (years)**		
17–25	161	11.5
26–35	1048	74.7
36–48	194	13.8
**Parity during pregnancy**		
None (unborn is first child)	645	49.0
One or two	619	47.0
Three or more	53	4.0
**Vocational degree**		
University (highest level)	482	34.1
Applied sciences (upper secondary level)	533	37.7
Secondary level (lower secondary level)	285	20.1
Vocational course(s)	17	1.2
No vocational education	68	4.8
Other	30	2.1
**Educational status (basic and vocational education)**		
1. Comprehensive school + lower vocational education (maximum)	221	16.0
2.Comprehensive school + higher vocational education or + degree from university of applied sciences, or high-school diploma + lower vocational education (maximum)level	245	17.7
3. High-school diploma + higher vocational education or + degree from university of applied sciences	438	31.6
4. Comprehensive school diploma or high-school diploma + university master’s degree or higher	481	34.7
**Educational status (used in analyses)**		
0 = Lower	466	33.7
1 = Higher	919	66.3
**Mother’s health**		
0 = Healthy	1098	78.1
1 = Not healthy	308	21.9

## Questionnaires

Toddlers’ social-emotional problems were measured at 24 months postpartum. At three and eight months, the questionnaires comprised the scale of the mother’s risk of a bonding disturbance with her baby and questionnaires about maternal stress, depression, and family atmosphere. The questions about the mother’s expectations of her baby, her relations with her partner and other adults, and demographic factors (e.g., age, education, parity, health) were included in the prenatal measurements.

### Prenatal measurements

***Prenatal bonding*** *was examined by measuring the* ***mothers’ expectations of their unborn babies*** using the Representations of Unborn Baby (RUB-M) scale. This is a self-reported 12-item Likert scale [34]. The parents answered the question *“What kind of expectations do you have of your future baby?”* They could choose from twelve statements, for example, *“I imagine […] that my baby will be satisfied and happy”*. A five-point response scale ranged from *“Not at all”* to *“I can’t say”* to *“Very much”*. We conducted a maximum likelihood factor analysis and extracted three factors: positive expectations of their relationship with the baby, negative expectations related to taking care of the baby, and positive expectations related to the baby’s regularity [13]. The factor scores were used in the analyses. (Table 2)

**Table 2:** Distributions of study variables

	Mean	SD	Min - Max	α
**Dependent factor**				
Social-emotional problems (BITSEA-score)	9.21	5.28	0.00 - 38.48	0.76
**Explanatory factors**				
PBQ—3 months	4.76	3.66	0.00–26.00	0.763
PBQ—8 months	3.91	3.49	0.00–42.00	0.781
Positive expectations related to relationship with baby	− 0.02	0.93	-6.91 − 0.82	Na
Negative expectations related to taking care of baby	0.01	0.83	-1.46–3.37	Na
Positive expectations related to regularity of baby	0.003	0.76	-2.03–2.20	Na
Closeness	4.06	0.60	1.80–5.00	0.697
Confidence	3.77	0.77	1.00–5.00	0.859
Anxiety	1.72	0.69	1.00–4.60	0.752
Prenatal family atmosphere	42.52	5.22	13.00–49.00	0.859
Postnatal family atmosphere (at 3 months)	42.21	5.73	15.00–49.00	0.861
Postnatal family atmosphere (at 8 months)	42.12	5.80	18.00–49.00	0.880
**Controlled variables**				
Prenatal depression	5.07	3.50	0.00–23.00	0.777
Prenatal stress	5.60	2.87	0.00–17.00	0.684

**Abbreviations**: SD Standard deviation, Min minimum, Max maximum, α Cronbach Alpha, Na Not applicable

***The mother’s attachment style within adult relationships (including that with spouse)*** was assessed using the Collins and Read (1990) Adult Attachment Scale (AAS), a self-report questionnaire comprising 18 items with a 1–5 scale. We calculated the three standardized subscales (closeness, confidence, and anxiety), as reported by Collins and Reed [35]. However, the closeness and anxiety in adult relationships subscales consisted of only five items because we excluded the item *“I am comfortable having others depend on me”* due to low communality and the improved reliability (Cronbach’s alpha) of the closeness subscale from 0.61 to 0.70. We also excluded the item “*I want to merge completely with another person”* due to the improved reliability of the anxiety subscale from 0.65 to 0.75. The confidence subscale comprised six items, for example “I am comfortable depending on others” with a reliability of 0.86. Higher values indicated a high level of closeness, confidence, or anxiety in adult relationships. (Table 2)

***Depression*** was measured using the ten-item version of the Center for Epidemiological Studies Depression Scale (CES-D, 36, 37). The response options ranged from 0 to 3 *(0 = rarely or not at all or less than once a week, 3 = all the time or 5–7 days per week)*. We calculated the total score, which ranged from 00.00 to 23.00 (α = 0.78, 10 items). Higher values indicated increased depression. (Table 2)

***Stress*** was measured using the five-item scale of the Global Measure of Perceived Stress (GMPS, 38) in which the options range from 0 to 4 *(0 = not even once, 4 = very often)*. We calculated the total score, which ranged from 00.00 to 17.00 (α = 0.68, 5 items). Higher values indicated increased stress. (Table 2)

### Measurements at three and eight months postpartum

***Mother–baby bonding disturbance*** was measured using Brockington’s Postpartum Bonding Questionnaire (PBQ), a widely used scale with good psychometric properties (31, 39 − 41). We used one of the four PBQ subscales, *general factor*, in this study. According to Brockington, this subscale *measures* “some kind of a problem in the mother–infant relationship” (40, p. 237). It comprises 12 items and has been reported to be reliable and valid [31, 39, 41]. We calculated the total score, as originally done by Brockington [40]. Higher values indicated more negative bonding. The alpha was 0.76 (at three months), and 0.78 (at eight months), and the range of the total score was 0–60 (Table 2).

***Family atmosphere*** was measured using a seven-item bipolar semantic differential scale, for example, *approving* (= 1) to *disapproving* (= 7), *safe* (= 1) to *unsafe* (= 7), *paralyzing* (= 1) to *enthusiastic* (= 7) and *reserved* (= 1) to *open* (= 7). Three of the items (approving–disapproving, safe–unsafe and approving–criticizing) were reverse coded. We calculated the total score, which ranged prenatally from 13.00 to 49.00 (α = 0.86), at three months postnatally from 15.00 to 49.00 (α = 0.86) and at eight months postnatally from 18 to 49.00 (α = 0.88). Higher values indicated a more positive family atmosphere. (Table 2)

***Depression*** and ***stress*** were measured at three and eight months, as in the prenatal phase.

### Measurements at 24 months postpartum

To measure the ***social-emotional problems*** of the two-year-old children, we used the 31-item Brief Infant-Toddler Social and Emotional Assessment (BITSEA, 42, 43), which measures social-emotional and behavioral problems (i.e., externalizing and internalizing problems and dysregulation, to e.g. overactivity, withdrawal, aggression, anxiety, etc.). We calculated the total score, which ranged from 0.00 to 38.48 (α = 0.76) (Table 2).

***The background variables*** were mother’s education, age, parity, and maternal health during pregnancy (Tables 1 and 2).

## Statistical analyses

We studied whether prenatal expectations of the unborn baby and the post-partum risk of bonding disturbances and other psycho-social factors (mother’s relationships with adults and family atmosphere) are associated with a child’s social-emotional symptoms at the age of two, using linear regression analysis. We conducted the modeling in four stages. *In the first stage*, we separately analyzed the association between each explanatory variable and social-emotional problems (i.e., the BITSEA total problem score), and took demographic factors (e.g., the mother’s age, parity, education, and health) into account. *In the second stage of the modeling*, we also controlled for prenatal depression. *In the third stage of the modeling*, we further controlled for prenatal stress. *In the fourth stage of the modelling*, we gathered depression and stress measures at the same timepoints as the explanatory variables. We used IBM SPSS Statistics 27 in all the statistical analyses.

## Results

### Description of the sample

This sample was a representative birth cohort. The mothers’ mean age was 30.7, which corresponds to the age of the women giving birth in the area. According to official statistics, the mean age of delivered women was 30.7 years [44]. Almost half of the mothers (N = 645, 49.0%) were expecting their first child. However, the women’s educational level in our data was higher than that of the general population: 71.8% of the mothers in our sample had a university or applied science degree, whereas in the general population, 25–40% of women have completed higher education. These percentages varied according to the residential district in which the women live (45, Table 1).

## Predictors of social-emotional problems at the age of two

First, we studied whether the prenatal expectations of the baby, postnatal bonding, the mother’s relationships with adults (including spouse) and family atmosphere were associated with the child’s social-emotional problems at the age of two when demographic factors were controlled for. According to linear regression analysis, all the studied explanatory factors, excluding the mother’s prenatal positive expectations of the regularity of the baby, were related to social-emotional problems in two-year-old children (Table 3). We found that impaired bonding at three (β = 0.259, p < 0.001) and eight months (β = 0.311, p < 0.001), as well as lack of positive expectations of relationship with baby (β = − 0.074, p < 0.05) and negative expectations related to taking care of baby (β = 0.109, p < 0.01) were related to increased social-emotional problems in two-year-old children. We also found that the mother’s lower closeness (β = − 0.183, p < 0.001), lower confidence (β = − 0.218, p < 0.001) and greater anxiety (β = 0.235, p < 0.001) in adult relationships (including relationship with spouse), as well as negative pre- and postnatal family atmosphere was significantly related to increased social-emotional problems in two-year-old children (prenatal: β = − 0.231, p < 0.001, p < 0.001; at three months postnatal: β = − 0.260, p < 0.001; at eight months postnatal: β = − 0.277, p < 0.001). Of the four covariates, only the mother’s parity (p = < 0.05) and age (p < 0.05) were significantly related to social-emotional problems when the child was two in many of the linear regression analyses. Children of younger mothers or with less siblings had more social-emotional problems at the age of two.

**Table 3:** Regression analysis of BITSEA with explanatory variables in the model, separately and controlled for demographics

Explanatory variables	β	S.E.	β std	t	p
**PBQ—3 months** Mother’s age Parity (1 = one or more) Education (1 = higher) Health (1 = not healthy)	0.363 − 0.073 − 0.396 − 0.015 0.597	0.048 0.044 0.191 0.400 0.417	0.259 − 0.060 − 0.073 − 0.001 0.049	7.582 − 1.652 − 2.072 − 0.038 1.433	< 0.001 0.099 0.039 0.970 0.152
**PBQ—8 months** Mother’s age Parity (1 = one or more) Education (1 = higher) Health (1 = not healthy)	0.433 − 0.080 − 0.433 − 0.049 0.337	0.048 0.044 0.191 0.404 0.421	. 0.311 − 0.065 − 0.080 − 0.004 0.027	9.101 − 1.801 − 2.271 − 0.121 0.801	< 0.001 0.072 0.023 0.903 0.424
**Positive expectations of relationship with baby** Mother’s age Parity (1 = one or more) Education (1 = higher) Health (1 = not healthy)	− 0.413 − 0.099 − 0.510 0.203 0.796	0.196 0.046 0.197 0.415 0.433	− 0.074 − 0.081 − 0.095 0.018 0.064	− 2.114 − 2.151 − 2.595 0.490 1.837	0.035 0.032 0.010 0.624 0.067
**Negative expectations related to taking care of baby** Mother’s age Parity (1 = one or more) Education (1 = higher) Health (1 = not healthy)	0.688 − 0.080 − 0.433 0.216 0.759	0.225 0.046 0.198 0.413 0.432	0.109 − 0.065 − 0.080 0.019 0.061	3.062 − 1.726 − 2.185 0.524 1.755	0.002 0.085 0.029 0.601 0.080
**Positive expectations related to regularity of baby** Mother’s age Parity (1 = one or more) Education (1 = higher) Health (1 = not healthy)	− 0.238 − 0.094 − 0.542 0.256 0.806	0.239 0.046 0.198 0.415 0.434	− 0.035 − 0.077 − 0.101 0.022 0.065	− 0.996 − 2.032 − 2.731 0.618 1.855	0.319 0.042 0.006 0.537 0.064
**High closeness** Mother’s age Parity (1 = one or more) Education (1 = higher) Health (1 = not healthy)	− 1.560 − 0.074 − 0.538 0.504 0.523	0.296 0.045 0.193 0.408 0.426	− 0.183 − 0.060 − 0.100 0.044 0.042	− 5.261 − 1.636 − 2.784 1.237 1.226	< 0.001 0.102 0.006 0.217 0.221
**High confidence** Mother’s age Parity (1 = one or more) Education (1 = higher) Health (1 = not healthy)	− 1.459 − 0.074 − 0.591 0.511 0.438	0.230 0.045 0.192 0.404 0.424	− 0.218 − 0.061 − 0.110 0.045 0.036	− 6.330 − 1.653 − 3.081 1.263 1.033	< 0.001 0.099 0.002 0.207 0.302
**High anxiety** Mother’s age Parity (1 = one or more) Education (1 = higher) Health (1 = not healthy)	1.873 − 0.075 − 0.615 0.688 0.545	0.275 0.045 0.191 0.406 0.421	0.235 − 0.061 − 0.114 0.060 0.044	6.813 − 1.678 − 3.212 1.696 1.295	< 0.001 0.094 0.001 0.090 0.196
**Positive family atmosphere (pre)** Mother’s age Parity (1 = one or more) Education (1 = higher) Health (1 = not healthy)	− 0.240 − 0.094 − 0.707 0.442 0.581	0.036 0.045 0.193 0.402 0.421	− 0.231 − 0.077 − 0.131 0.039 0.047	− 6.748 − 2.103 − 3.670 1.099 1.381	< 0.001 0.036 0.000 0.272 0.168
**Positive family atmosphere (3 months)** Mother’s age Parity (1 = one or more) Education (1 = higher) Health (1 = not healthy)	− 0.235 − 0.093 − 0.735 0.376 0.500	0.031 0.044 0.192 0.399 0.418	− 0.260 − 0.076 − 0.136 0.033 0.041	− 7.609 − 2.095 − 3.827 0.942 1.197	< 0.001 0.037 0.000 0.346 0.232
**Positive family atmosphere (8 months)** Mother’s age Parity (1 = one or more) Education (1 = higher) Health (1 = not healthy)	− 0.243 − 0.108 − 0.685 0.319 0.339	0.030 0.045 0.192 0.405 0.425	− 0.277 − 0.089 − 0.127 0.028 0.027	− 8.054 − 2.424 − 3.569 0.789 0.797	< 0.001 0.016 0.000 0.430 0.426

Multicollinearity: VIF > 0.1; Tolerance < 10

In the next stage, we controlled for the mother’s prenatal depression alongside the other demographics. We found that their mother’s prenatal depression (p = < 0.001), parity (p = < 0.01) and age (p = < 0.05) were significantly related to social-emotional problems among two-year-olds. In these models, the increased social-emotional problems of two-year-olds were still related to the mother’s negative expectations related to taking care of her baby (β = 0.072, p < 0.05), impaired bonding at three (β = 0.167, p < 0.001) and at eight months postnatally (β = 0.230, p < 0.001), mother’s more negative adult relationships (closeness: β = − 0.078, p < 0.05; confidence: β = − 0.088, < 0.05; anxiety: β = 0.105, p < 0.01), and negative family atmosphere prenatally (β = − 0.102, p < 0.01) at three months (β = − 0.153, p < 0.001) and eight months (β = − 0.177, p < 0.001) postnatally (Supplement 1). The associations between mother–baby bonding and family atmosphere and the child’s psycho-social symptoms remained significant even when depression was controlled for at the same timepoint as the explanatory factors (Supplement 2).

Finally, we also adjusted for prenatal stress in addition to the demographic factors. We found that all the results remained significant in the linear regression models with stress and demographic factors as covariates. First, the negative prenatal expectations related to taking care of one’s baby (β = 0.074, p < 0.05), and the mother’s risk of bonding disturbance at three months (β = 0.189, p < 0.001) and eight months (β = 0.249, p < 0.001) were significantly related to increased social-emotional problems at two years. Second, stress (p < 0.001) and parity (p = < 0.01) were also significantly related to the social-emotional problems of two-year-old children in all models (Supplement 2). Finally, the mother’s more negative adult relationships (closeness:β = − 0.112, p < 0.01; confidence: β = − 0.130, p < 0.01; anxiety: β = 0.147, p < 0.001), and a more negative family atmosphere prenatally (β = − 0.142, p < 0.001), and at three months (β = − 0.173, p < 0.001) and eight months postnatally (β =− 0.202, p < 0.001) were significantly related to increased social-emotional problems when the child was two years old (Supplement 3). The significance of mother–baby bonding and family atmosphere remained even after stress was controlled for at the same timepoint as the explanatory factors (Supplement 4).

## Discussion

So far, the link between perinatal mother–baby bonding and a child’s social-emotional development has been under-investigated. There is also a consensus on the need for further research on the development of prenatal bonding after childbirth and the association between perinatal bonding and a child’s developmental outcomes [4–6, 12].

Our study adds to the current literature by investigating the role of perinatal bonding and maternal relationships within and outside the family in a child’s social-emotional development in a longer follow-up study setting than most previous studies, and by controlling for mother’s depression, stress, and demographic factors. Our study further responds to the request to study bonding using new measures (4, p. 15). Our main findings are that both perinatal bonding and a mother’s better adult relationships within and outside the family are strongly related to a child’s better social-emotional development.

We found that a mother’s more negative prenatal expectations of their relationship with their baby and negative expectations related to taking care of their baby are related to social-emotional problems in two-year-old children. The mother’s negative expectations related to taking care of her baby remained significant even after controlling for prenatal depression and stress. Our findings are similar to those reported by Alhusen et al., who found [1] that better prenatal bonding is related to a child’s better development (i.e., gross and fine motor skills, communication, problem-solving, and personal-social factors) at the age of 14–26 months. However, they also reported that the association between prenatal bonding and the mother’s anxious attachments and developmental problems became insignificant when the mother’s avoidant attachment style and postnatal depression were taken into account. Our findings are contradictory to these, because in our study, the association between prenatal bonding (i.e., negative expectations related to taking care of the baby) and social-emotional development remained significant even after controlling for prenatal depression. However, we did not control for the mother’s avoidant attachment style. The study designs and measures (i.e. timing of the measurement, measures of developmental outcomes, maternal attachment style and prenatal bonding) also differed to some extent, which may partly explain the somewhat differing results. We measured *social-emotional problems* using the BITSEA, whereas Alhusen et al. [1] used the Ages and Stages Questionnaire, which includes many other developmental factors in addition to social emotional. Regarding *prenatal bonding*, we measured the maternal expectations of the baby [34], and used The Representations of Unborn Baby Scale (RUB-M), but Alhusen et al. [1] used The Maternal-Fetal Attachment Scale (MFA).

Moreover, our findings suggest that not only prenatal expectations of the baby, but also postnatal bonding is a significant risk factor for child development. We found that bonding difficulties with three- and eight-month-old babies was associated with more social-emotional problems in two-year-old children even after controlling for the mother’s depression and stress separately, both prenatally and at three and eight months postnatally, using demographic factors (mother’s age, parity, education, and health). Our results are similar to the recent findings of Le Bas et al., who reported [5] that both pre- and postnatal bonding are associated with a child’s social-emotional development (including social-emotional competence, independence, emotional stability, and social approach behavior), and behavioral development. However, our research setting differed to some extent from theirs. In our study, the children were two years old at the end of our follow-up, whereas in Le Bas et al.’s study [5], the children were one. Moreover, we followed bonding development from pregnancy to three and eight months postpartum (three times), whereas Le Bas et al. [5] assessed bonding five times from the beginning of pregnancy to 12 months postnatally.

Our findings regarding the association between maternal bonding and a child’s social-emotional development are also consistent with those of Behrendt et al. They reported [2] that a mother’s sensitivity at six to eight months is associated with less social-emotional and behavioral problems (measured by BITSEA), and that postnatal bonding at six to eight months is associated with social-emotional competence (e.g., prosocial behavior, attention abilities, empathy, awareness of emotions in other people) and fewer problems at 12–16 months of age. Postnatal depression and maternal emotion regulation were taken into account in the study. These results highlighted the mother’s role in the mother–child interaction and the child’s psychosocial development, and are in line with our findings.

Le Bas et al. [4] reviewed 15 studies of mother–baby pre- and postnatal bonding disturbances and children’s developmental outcomes. Bonding was associated with the baby having a higher attachment quality, lower colic rating, easier temperament, and positive infant mood. However, only three of the reviewed studies reported social emotional outcomes (i.e., explorative activity, negative and positive effects, solicitation of attention, social-emotional competence, externalizing and internalizing behaviors) and found weak evidence for the association between pre- and postnatal bonding and social-emotional development. Our results lend further support to Le Bas et al.’s conclusion [4] that prenatal bonding relates to social-emotional development at the age of two. In their meta-analysis, Le Bas et al. reported (4, p.15) weak evidence that better maternal bonding is associated with better developmental outcomes in infants aged 0–24 months. Our findings add to those of previous studies by suggesting a strong association between maternal bonding and a child’s development.

As both the pre- and postnatal bonding in our study were associated with the child’s developmental outcomes, their cumulative influence on bonding may explain our results (see, e.g., 5, p. 9). For example, De Cock et al. reported [29, 46] that weaker prenatal bonding predicted weaker postnatal bonding, which in turn, predicted problems in the child’s executive functioning at the age of two. The association between pre- and postnatal bonding in our sample has been reported earlier [9], which suggests that bonding could cumulatively influence a child’s development.

We also found that a mother’s good relationships with her spouse and other adults, (i.e., the mother’s ability to form close or confident adult relationships without anxiety), were related to better social emotional development of two-years-olds. This finding remained significant even after controlling for prenatal depression and stress using demographic factors, suggesting that good maternal adult relationships may be beneficial in terms of a child’s social-emotional development. Our results are in concordance with the findings of Junge et al., who reported [17] that poor social support of the mother in the perinatal phase is related to more social-emotional problems in the child. Another previous study reported that the support of a spouse was most important for mothers. Parfitt Ayers, Pike, Jessop, & Ford claimed [47] that the relationship between the parents during pregnancy is the most important factor in terms of postnatal bonding (3 months and 15 months) and a child’s positive development.

In our study, we measured maternal adult relationships using the Adult Attachment Scale, making comparisons with studies that measured maternal attachment style appropriate. From this point of view, our results are in accordance with those of Alhusen et al., who reported [1] that securely attached mothers had better prenatal bonding and lower postnatal depressiveness, which in turn was associated with better developmental outcomes (including social-emotional development) when the child was 14–26 months old. Priel and Besser’s findings also support our results. According to them [48], mothers with a secure attachment style had easier perceptions of their babies than mothers with an insecure style. They also found [48] that prenatal bonding was mediated by a maternal romantic attachment style which, in turn, is inversely related to maternal perceptions of a four-month-old child’s temperamental difficulties (i.e., to rhythmicity, approach, adaptability, intensity, and mood).

We found that both a pre- and postnatal negative family atmosphere were related to a child’s social-emotional problems at the age of two. The results remained significant after controlling for the mother’s depression and stress separately, pre- and postnatally, at three and eight months using demographic factors. This suggests that a negative family atmosphere during the perinatal phase is an independent psycho-social risk factor alongside stress and depression, even though they are reported to be inter-related [49]. We found respective studies that had examined family atmosphere as an explanatory factor several times, pre- and postnatally. However, based on previous studies [2, 29] we can summarize that a mother receiving support seems to be related to pre- and postnatal bonding [29], which in turn is associated with a child’s better developmental social-emotional outcomes [2]. According to Huang, Costeines and Kaufman [50], an adolescent mother’s higher stress and lower social support at six months postpartum was associated with higher maternal depression at six months postpartum, which in turn, was associated with developmental delays (in which social-emotional development is one factor of the measure) at 18 months postpartum.

As expected, both depression and stress (prenatally and at three and eight months) were related to a child’s psychosocial symptoms at the age of two, which is consistent with several previous studies, according to which the mother’s depression and stress play a major role in the mother–baby interaction [25, 30, 51, 52] and mother–baby bonding [14]. Moreover, a higher level of or more persistent depressive symptoms in mothers in the perinatal phase is associated with increased emotional problems among children after birth [15, 17–19].

The effect of maternal postnatal depression on a child’s development has been considered interactive. A depressed mother may communicate with her baby less actively, for example, her parenting may include less touching of the infant or less infant-directed speech [53]. If the adult on whom the child is dependent is unable, for some reason [e.g., due to depression or stress during pregnancy [51, 52], or lack of support [17, 47]], to be psychologically available, responsive and sensitive to the extent the child expects, the child may develop an insecure attachment style [11], which they then implement in different relationships with other significant adults and peers [11]. Since bonding may be part of the broader attachment construct [1, 6], the continuation and cumulative influence of prenatal weak bonding [54] on the postnatal phase and its significance for the child’s social-emotional problems becomes understandable.

Bonding problems may implicate problems in parenting skills [i.e., less engagement, flexibility, sensitivity, and warmth (55, p. 36)], which in turn may affect a child’s developmental outcomes [56]. Negative parenting, especially in conflict situations, is known to impair a child’s outcomes. For example, Scaramella, Sohr-Preston, Mirabile, Robinson and Callah found [57] that a mother’s harsh responses to her 12-month-old child’s disobedience increased the child’s distress at the age of two. Providing early enough support for parenting and interacting with one’s baby is immensely important, because the consequences of negative parenting have been found to continue into later childhood [58].

In addition to parenting problems, a child’s characteristics may also be associated with bonding. The child’s increased demands for maternal care immediately after birth can be understood from a neurobiological perspective. Maternal prenatal depression is assumed to be associated with the mother’s elevated cortisol levels, which are transmitted via the placenta to the unborn baby [52]. Cortisol levels in infants of depressed mothers have been found to be higher than those in infants of non-depressed mothers, and due to the exposure to excess maternal cortisol, infants can be more sensitive to postnatal stressor factors [51]. This might explain the effect of prenatal psychological risk factors on not only the mother but also on the baby, thus making it a weakening factor in parenting. Because in our study, both impaired prenatal bonding and prenatal depression were equally significant to the child’s social-emotional development, the influence of neurobiological mechanisms may be a part of the development of poorer bonding [9] and the child’s social-emotional problems.

All in all, to understand the dynamics of our results, we must look at the association between bonding, maternal adult relationships, psychological risk factors, and social-emotional outcomes all together. Based on these pre- and postnatal psycho-biological and interactive mechanisms, as well as on their continuity and accumulation (see 5, 54), we suggest that both a baby’s sensitivity to stress (see 25) and the parent–child interaction together mediate the negative effects of parental depression or stress on the child’s social-emotional development.

As the baby’s demands on the mother increase as a result of the mother’s perinatal psychological and communicative problems, the mother needs extra support and help to take care of her baby, and if she does not get this, her resources dwindle, which in turn further impairs maternal bonding [9] and the two-year-old’s social-emotional development [17]. Alhusen et al., and Kingston, Tough and Whitfield recommend [1, 59] developing appropriate intervention programs for mothers at risk, aiming to, for example, strengthen their relationship with the home-visiting nurse, and thereby develop self-efficacy in parenting skills as a first-time mother. Some intervention studies have already been conducted, and the results are promising. In addition, according to Sierau, Dähne, Brand, Kurtz, von Klitzing and Jungmann [60], mothers who were at risk benefitted from a home visit program in which their maternal capacity as a mother, their social support, and their knowledge of childcare improved.

Of the demographic factors (i.e., mother’s education, age, parity, health) only the mother’s age and parity were significantly associated with the child’s social-emotional development. First-born children and children with younger mothers were at a higher risk of having social-emotional problems at the age of two. This result could be related to mothering skills, which improve with experience in childcare [54, 61].

The effects of maternal bonding and psycho-social factors on a child’s social-emotional development were independent of the mother’s education and health. In previous studies, a mother’s high education level was related to both less behavioral problems in three-year-olds [62] and to better postnatal bonding [63], but paradoxically, also to low levels of bonding [29]. The difference between these results can be explained by the fact that our data were collected in a country in which social-economic differences are generally small and education and the health and welfare system are good. For example, infant health check-ups with the same nurse take place almost monthly in the first year of the child’s life, and most mothers receive advice in maternity clinics [64]. In addition, maternity and parental leaves are available to all families, and this supports the development of mother–baby relationships [65, 66].

## Limitations

The study was based on a large, representative birth cohort. It comprised young families with relatively low rates of psychosocial problems. It included rather highly educated parents, and lower social-economic groups were under-represented. Our study followed children from the last trimester of pregnancy to two years of age. The maternal emotional bond with the baby before birth was not examined using a standardized measure, but our results are supported by the longitudinal research results of Le Bas et al. [5], which verify the validity of measuring prenatal bonding. The dropout rate at 24 months was relatively high, which may limit the generalizability of the findings if this was related to explanatory factors or outcomes. However, dropout did not affect our results or conclusions regarding the associations between bonding and a child’s social emotional development. The missing data analysis showed that at both three and eight months, the respondents’ bonding disturbance **scores** were higher than those of the participants who dropped out (at 3 months: t = -2.84, p = 0.005; at 8 months t = -2.38, p = 0.018), which shows that the results are not due to the disappearance of mothers with bonding problems from the longitudinal data. Moreover, it is worth noticing that in this sample, the number of mothers at risk of bonding disturbances was quite low at both three (n = 66) and eight months (n = 36). Therefore, further studies with larger sample sizes or with case-control settings are needed to confirm our findings.

## Summary

In this longitudinal study, we examined how maternal bonding and psycho-social factors are associated with social-emotional problems in two-year-old children. Our data came from a birth cohort from which data were collected at four timepoints: prenatally during the third trimester, and postnatally at 3, 8 and 24 months. Our study adds to the current literature by investigating the role of perinatal bonding and maternal relationships within and outside the family in a child’s social-emotional development in a longer follow-up study setting than most previous studies, and by controlling for mother’s depression, stress, and demographic factors. In our study, better pre- and postnatal bonding, and better interpersonal relationships within and outside the family were associated with better social-emotional development, and maternal depression and stress in turn were associated with weaker social-emotional outcomes. Moreover, we found that perinatal family atmosphere and the mother’s prenatal relationships with adults and her spouse were associated with two-year-olds’ social-emotional development. Therefore, we emphasize that not only the mother’s psychological state, but also her emotional commitment to her unborn baby is significant. The findings emphasize the importance of recognizing problems in the evolving mother–baby relationship already during pregnancy in order to develop intervention programs to reduce mothers’ psychosocial problems and to strengthen maternal bonding with suitable support, which in turn, could have a positive impact on the child’s social-emotional development. It is also important to follow how bonding develops during the first year after birth, as well as to evaluate the quality of the mother’s relationships within and outside the family in addition to psychiatric symptoms.

### Electronic supplementary material

Supplementary Material
