# Supplementary material for: A Longitudinal Study of Maternal Postnatal Bonding and Psychosocial Factors that Contribute to Social-Emotional Development
Source: Child Psychiatry Hum Dev. 2022 Jul 23;55(1):274–86. doi: 10.1007/s10578-022-01398-5 (PMC10796530; doi:10.1007/s10578-022-01398-5)
Supplement: Supplementary file 1 — Supplementary Material [file 10578_2022_1398_MOESM1_ESM.docx]

**Supplement 1.** Regression analysis of BITSEA with explanatory variables in the model, separately and controlled for demographics and prenatal depression

| **Explanatory variables** | **β** | **S.E.** | **β std** | **t** | **p** |
| --- | --- | --- | --- | --- | --- |
| **PBQ—3 months**  Mother’s age  Parity (1=one or more)  Education (1=higher)  Health (1=not healthy)  Prenatal depression | .235  -.073  -.555  .266  .327  .366 | .051  .043  .188  .393  .409  .055 | .167  -.060  -.103  .023  .027  .238 | 4.648  -1.698  -2.950  .679  .799  6.614 | <.001  .090  .003  .498  .425  <.001 |
| **PBQ—8 months**  Mother’s age  Parity (1=one or more)  Education (1=higher)  Health (1=not healthy)  Prenatal depression | .321  -.076  -.566  .185  .162  .339 | .050  .043  .188  .398  .413  .055 | .230  -.062  -.104  .016  .013  .220 | 6.415  -1.748  -3.009  .464  .392  6.151 | <.001  .081  .003  .643  .695  <.001 |
| **Positive expectations of relationship with baby**  Mother’s age  Parity (1=one or more)  Education (1=higher)  Health (1=not healthy)  Prenatal depression | -.048  -.087  -.660  .463  .434  .458 | .192  .044  .189  .398  .417  .053 | -.009  -.072  -.122  .041  .035  .297 | -.248  -1.987  -3.488  1.162  1.040  8.590 | .804  .047  .001  .246  .299  <.001 |
| **Negative expectations related to taking care of baby**  Mother’s age  Parity (1=one or more)  Education (1=higher)  Health (1=not healthy)  Prenatal depression | .455  -.076  -.600  .429  .404  .448 | .217  .044  .191  .396  .416  .052 | .072  -.062  -.111  .038  .033  .291 | 2.099  -1.727  -3.148  1.083  .970  8.559 | .036  .084  .002  .279  .332  <.001 |
| **Positive expectations related to regularity of baby**  Mother’s age  Parity (1=one or more)  Education (1=higher)  Health (1=not healthy)  Prenatal depression | -.016  -.087  -.663  .471  .434  .461 | .230  .044  .190  .397  .417  .052 | -.002  -.071  -.123  .041  .035  .299 | -.069  -1.972  -3.487  1.186  1.039  8.792 | .945  .049  .001  .236  .299  <.001 |
| **High closeness**  Mother’s age  Parity (1=one or more)  Education (1=higher)  Health (1=not healthy)  Prenatal depression | -.667  -.076  -.671  .580  .296  .418 | .310  .044  .188  .395  .414  .056 | -.078  -.062  -.124  .051  .024  .273 | -2.151  -1.745  -3.571  1.470  .715  7.524 | .032  .081  <.001  .142  .475  <.001 |
| **High confidence**  Mother’s age  Parity (1=one or more)  Education (1=higher)  Health (1=not healthy)  Prenatal depression | -.586  -.077  -.687  .565  .285  .397 | .259  .044  .188  .394  .414  .059 | -.088  -.063  -.127  .050  .023  .259 | -2.260  -1.765  -3.659  1.435  .690  6.687 | .024  .078  <.001  .152  .491  <.001 |
| **High anxiety**  Mother’s age  Parity (1=one or more)  Education (1=higher)  Health (1=not healthy)  Prenatal depression | .838  -.077  -.695  .648  .332  .380 | .315  .044  .187  .396  .412  .060 | .105  -.063  -.129  .057  .027  .248 | 2.661  -1.760  -3.708  1.635  .805  6.319 | .008  .079  <.001  .102  .421  <.001 |
| **Positive family atmosphere (pre. continuing factor)**  Mother’s age  Parity (1=one or more)  Education (1=higher)  Health (1=not healthy)  Prenatal depression | -.106  -.085  -.735  .538  .348  .384 | .041  .044  .188  .393  .412  .060 | -.102  -.070  -.136  .047  .028  .250 | -2.610  -1.959  -3.906  1.369  .843  6.408 | .009  .050  <.001  .171  .399  <.001 |
| **Positive family atmosphere (3 months. continuing factor)**  Mother’s age  Parity (1=one or more)  Education (1=higher)  Health (1=not healthy)  Prenatal depression | -.139  -.087  -.762  .510  .285  .356 | .034  .043  .188  .391  .411  .058 | -.153  -.071  -.141  .045  .023  .232 | -4.080  -1.993  -4.055  1.304  .693  6.185 | <.001  .047  <.001  .193  .489  <.001 |
| **Positive family atmosphere (8 months. continuing factor)**  Mother’s age  Parity (1=one or more)  Education (1=higher)  Health (1=not healthy)  Prenatal depression | -.155  -.096  -.745  .469  .212  .348 | .033  .044  .188  .397  .418  .058 | -.177  -.079  -.138  .041  .017  .225 | -4.723  -2.194  -3.957  1.182  .507  6.035 | <.001  .029  <.001  .238  .612  <.001 |

Multicollinearity: VIF>0.1; Tolerance < 10

**Supplement 2.** Linear regression analysis of BITSEA with explanatory variables, separately and controlled for demographics and depression measured at same timepoint as explanatory variables

| **Explanatory variables** | **β** | **S.E.** | **β std** | **t** | **p** |
| --- | --- | --- | --- | --- | --- |
| PBQ—3 months  Mother’s age  Parity (1=one or more)  Education (1=higher)  Health (1=not healthy)  Depression—3 months | .186  -.058  -.545  .217  .355  .346 | .054  .043  .188  .393  .410  .054 | .133  -.047  -.101  .019  .029  .246 | 3.431  -1.328  -2.897  .552  .865  6.385 | .001  .185  .004  .581  .387  <.001 |
| PBQ—8 months  Mother’s age  Parity (1=one or more)  Education (1=higher)  Health (1=not healthy)  Depression—8 months | .303  -.080  -.536  .204  .190  .237 | .054  .044  .191  .402  .417  .050 | .218  -.065  -.099  .018  .015  .185 | 5.566  -1.818  -2.810  .506  .456  4.739 | <.001  .069  .005  .613  .649  <.001 |
| Positive family atmosphere—3 months  Mother’s age  Parity (1=one or more)  Education (1=higher)  Health (1=not healthy)  Depression—3 months | -.110  -.070  -.711  .425  .309  .352 | .036  .044  .188  .391  .411  .056 | -.122  -.057  -.132  .037  .025  .250 | -3.046  -1.597  -3.786  1.089  .752  6.288 | .002  .111  <.001  .276  .452  <.001 |
| Positive family atmosphere—8 months  Mother’s age  Parity (1=one or more)  Education (1=higher)  Health (1=not healthy)  Depression—8 months | -.140  -.099  -.698  .471  .246  .249 | .038  .044  .190  .402  .421  .055 | -.159  -.081  -.129  .041  .020  .195 | -3.684  -2.220  -3.668  1.174  .584  4.509 | <.001  .027  <.001  .241  .560  <.001 |

Multicollinearity: VIF>0.1; Tolerance < 10

**Supplement 3.**  Regression analysis of BITSEA with explanatory variables in the model, separately and controlled for demographics and prenatal stress

| **Explanatory variables** | **β** | **S.E.** | **В stdz** | **t** | **p** |
| --- | --- | --- | --- | --- | --- |
| **PBQ—3 months**  Mother’s age  Parity (1=one or more)  Education (1=higher)  Health (1=not healthy)  Prenatal stress | .266  -.066  -.535  .147  .401  .385 | .049  .043  .189  .392  .409  .064 | .189  -.054  -.100  .013  .033  .210 | 5.389  -1.524  -2.840  .375  .979  6.001 | <.001  .128  .005  .708  .328  <.001 |
| **PBQ—8 months**  Mother’s age  Parity (1=one or more)  Education (1=higher)  Health (1=not healthy)  Prenatal stress | .345  -.075  -.541  .090  .220  .343 | .049  .044  .189  .397  .414  .065 | .249  -.062  -.100  .008  .018  .187 | 7.029  -1.718  -2.867  .226  .531  5.295 | <.001  .086  .004  .822  .595  <.001 |
| **Positive expectations of relationship with baby**  Mother’s age  Parity (1=one or more)  Education (1=higher)  Health (1=not healthy)  Prenatal stress | -.104  -.082  -.636  .355  .510  .480 | .192  .044  .191  .400  .420  .064 | -.019  -.067  -.118  .031  .041  .263 | -.541  -1.845  -3.335  .888  1.215  7.551 | .589  .065  .001  .375  .225  <.001 |
| **Negative expectations related to taking care of baby**  Mother’s age  Parity (1=one or more)  Education (1=higher)  Health (1=not healthy)  Prenatal stress | .466  -.070  -.577  .333  .479  .471 | .218  .045  .192  .398  .419  .063 | .074  -.058  -.108  .029  .039  .258 | 2.135  -1.575  -3.005  .836  1.144  7.519 | .033  .116  .003  .403  .253  <.001 |
| **Positive expectations related to regularity of baby**  Mother’s age  Parity (1=one or more)  Education (1=higher)  Health (1=not healthy)  Prenatal stress | -.171  -.079  -.656  .362  .501  .486 | .230  .044  .192  .399  .420  .062 | -.025  -.065  -.122  .032  .041  .266 | -.744  -1.779  -3.422  .909  1.193  7.791 | .457  .076  .001  .364  .233  <.001 |
| **High closeness**  Mother’s age  Parity (1=one or more)  Education (1=higher)  Health (1=not healthy)  Prenatal stress | -.953  -.068  -.649  .505  .335  .431 | .301  .044  .189  .396  .415  .065 | -.112  -.056  -.121  .045  .027  .235 | -3.170  -1.541  -3.438  1.274  .806  6.677 | .002  .124  .001  .203  .421  <.001 |
| **High confidence**  Mother’s age  Parity (1=one or more)  Education (1=higher)  Health (1=not healthy)  Prenatal stress | -.868  -.071  -.667  .495  .319  .388 | .249  .044  .188  .395  .415  .068 | -.130  -.058  -.124  .044  .026  .212 | -3.488  -1.611  -3.540  1.253  .769  5.681 | .001  .107  <.001  .211  .442  <.001 |
| **High anxiety**  Mother’s age  Parity (1=one or more)  Education (1=higher)  Health (1=not healthy)  Prenatal stress | 1.168  -.072  -.677  .609  .389  .370 | .301  .044  .188  .397  .413  .069 | .147  -.059  -.126  .054  .032  .202 | 3.885  -1.636  -3.600  1.531  .942  5.373 | <.001  .102  <.001  .126  .346  <.001 |
| **Positive family atmosphere (pre)**  Mother’s age  Parity (1=one or more)  Education (1=higher)  Health (1=not healthy)  Prenatal stress | -.147  -.083  -.733  .453  .413  .372 | .039  .044  .189  .393  .413  .069 | -.142  -.068  -.136  .040  .034  .203 | -3.747  -1.897  -3.882  1.151  .998  5.374 | <.001  .058  <.001  .250  .318  <.001 |
| **Positive family atmosphere (3 months)**  Mother’s age  Parity (1=one or more)  Education (1=higher)  Health (1=not healthy)  Prenatal stress | -.157  -.084  -.755  .424  .367  .359 | .033  .044  .189  .391  .411  .068 | -.173  -.069  -.140  .037  .030  .196 | -4.683  -1.915  -4.001  1.083  .891  5.297 | <.001  .056  <.001  .279  .373  <.001 |
| **Positive family atmosphere (8 months)**  Mother’s age  Parity (1=one or more)  Education (1=higher)  Health (1=not healthy)  Prenatal stress | -.177  -.097  -.735  .384  .256  .348 | .032  .044  .189  .397  .418  .067 | -.202  -.080  -.136  .034  .021  .190 | -5.540  -2.204  -3.890  .968  .612  5.207 | <.001  .028  <.001  .334  .541  <.001 |

Multicollinearity: VIF>0.1; Tolerance < 10

**Supplement 4** Linear regression analysis of BITSEA with explanatory variables, separately and controlled for demographics and stress measured at same timepoint as explanatory variables

| Explanatory variables | β | Std. Error | В (Stdz) | t | p |
| --- | --- | --- | --- | --- | --- |
| PBQ—3 months  Mother’s age  Parity (1=one or more)  Education (1=higher)  Health (1=not healthy)  Stress—3 months | .231  -.061  -.604  .200  .378  .330 | .054  .044  .191  .396  .412  .062 | .165  -.050  -.112  .018  .031  .203 | 4.302  -1.400  -3.156  .505  .919  5.290 | <.001  .162  .002  .613  .358  <.001 |
| PBQ—8 months  Mother’s age  Parity (1=one or more)  Education (1=higher)  Health (1=not healthy)  Stress—8 months | .318  -.077  -.608  .236  .252  .278 | .053  .044  .192  .404  .416  .060 | .229  -.063  -.112  .021  .020  .178 | 6.008  -1.755  -3.164  .584  .606  4.645 | <.001  .080  .002  .560  .545  <.001 |
| Positive family-atmosphere—3 months  Mother’s age  Parity (1=one or more)  Education (1=higher)  Health (1=not healthy)  Stress—3 months | -.143  -.074  -.813  .451  .320  .334 | .035  .044  .189  .393  .412  .063 | -.158  -.061  -.151  .040  .026  .207 | -4.102  -1.703  -4.294  1.149  .775  5.329 | <.001  .089  <.001  .251  .439  <.001 |
| Positive family-atmosphere—8 months  Mother’s age  Parity (1=one or more)  Education (1=higher)  Health (1=not healthy)  Stress—8 months | -.151  -.096  -.789  .518  .303  .295 | .036  .044  .191  .402  .420  .064 | -.171  -.078  -.146  .045  .025  .189 | -4.195  -2.159  -4.128  1.288  .722  4.603 | <.001  .031  <.001  .198  .470  <.001 |

Multicollinearity: VIF>0.1; Tolerance < 10
